# Supplementary material for: Association study of depressive symptoms and periodontitis in an obese population: Analysis based on NHANES data from 2009 to 2014
Source: PLoS One. 2024 Dec 19;19(12):e0315754. doi: 10.1371/journal.pone.0315754 (PMC11658469; doi:10.1371/journal.pone.0315754)
Supplement: Attachment — Submitted filename: Response to Reviewers.docx [file pone.0315754.s001.docx]

**Response to Isabel Cristina Gonçalves Leite：**

1. A rebuttal letter that responds to each point raised by the academic editor and reviewer(s). You should upload this letter as a separate file labeled 'Response to Reviewers'.

This document details our responses to each point raised by the reviewers and the editor. For any suggested revisions, we have outlined our corresponding changes to the manuscript or explanations where necessary.

2. A marked-up copy of your manuscript that highlights changes made to the original version. You should upload this as a separate file labeled 'Revised Manuscript with Track Changes'.

A version of the manuscript containing tracked changes is included to clearly highlight all modifications made in response to the reviewers’ comments.

3. An unmarked version of your revised paper without tracked changes. You should upload this as a separate file labeled 'Manuscript'.

A clean, unmarked version of the revised manuscript is provided for your convenience.

**Response to Journal Requirements：**

1. Please ensure that your manuscript meets PLOS ONE's style requirements, including those for file naming.

We have carefully reviewed and ensured that the manuscript adheres to PLOS ONE’s formatting requirements, including file naming conventions.

If there are still any formatting issues, please let us know, and we will promptly address them.

2. Please state what role the funders took in the study.

Thank you for pointing out the required amendment to the funding statement. We have updated the statement as follows:

“This study was funded by the Health Commission of Hebei Province (20240102). The funders had no role in study design, data collection and analysis, decision to publish, or preparation of the manuscript.”

This revised funding statement has also been included in our cover letter. Please inform us if further revisions are needed.

3. Please note that your Data Availability Statement is currently missing the repository name.

We noted the absence of the repository name in the Data Availability Statement and have corrected it as follows:

"Publicly available datasets were analyzed in this study, specifically from the National Health and Nutrition Examination Survey (NHANES). These datasets can be accessed at www.cdc.gov/nchs/nhanes/."

4. We note you have included a table to which you do not refer in the text of your manuscript. Please ensure that you refer to Table 1 in your text; if accepted, production will need this reference to link the reader to the Table.

We have reviewed the manuscript and added a reference to Table 1 in the main text to ensure consistency between the table and the manuscript content. This change has been highlighted in red text in the 'Revised Manuscript with Track Changes' document.

**Response to “ 5. Review Comments to the Author”：**

**Reviewer #1:**

1. Explanation of Unexpected Results:

First and foremost, we extend our heartfelt thanks for your meticulous review of our manuscript and the insightful suggestions you have offered. In response to your feedback, we acknowledge the need for a more thorough discussion on how obesity impacts periodontitis through adipokines, particularly in the discussion of adipokines such as leptin and adiponectin. Consequently, we have re-examined a wealth of literature and have added references to adipokines, including leptin and adiponectin, in the fifth paragraph of the discussion section.

2. Consideration of Additional Comparison Groups:

Thank you for your insightful comments on our manuscript titled "Association Study of Depression and Periodontitis in an Obese Population: Analysis Based on NHANES Data from 2009 to 2014." We appreciate your recognition of the focused nature of our study population, which is indeed centered on individuals with obesity.

We acknowledge that our targeted research design may limit the generalizability of our findings. However, our choice to focus on this specific demographic is deliberate, given the established links between obesity and a myriad of health issues, including depression and periodontitis, as well as the growing global prevalence of obesity. Our aim is to provide deeper insights into this high-risk group and to contribute targeted data for future research and clinical practice.

We are grateful for your constructive suggestion and are committed to refining our participant categorization in forthcoming studies to more precisely elucidate the relationship between depression, obesity, and periodontitis. In our next research phase, we will aim to implement more detailed groupings to better understand the complex dynamics at play. Your feedback is invaluable in helping us shape our research approach, and we are dedicated to clarifying the interconnections between these health conditions as per your recommendations.

We appreciate your expertise and look forward to incorporating these insights into our future work.

3. Terminology Consistency:

We appreciate your keen observation regarding the terminology used in our study. We agree that it is essential to maintain consistency in referring to participants as having "depressive symptoms" rather than "depressed" to avoid implying a clinical diagnosis. We have updated our document to reflect this change, replacing all instances of "depressed symptoms" and "depression symptoms" with "depressive symptoms." Thank you for your constructive suggestion.

4. Limitations of the PHQ-9 Questionnaire:

We sincerely appreciate your insightful comments. We have addressed the limitations of PHQ-9 in capturing transient emotional states rather than chronic depressive disorders in the seventh paragraph of the discussion. Your suggestion is highly valuable, and we acknowledge the complexity of diagnosing depression. We will certainly consider incorporating longitudinal measures and clinical diagnostic tools in future studies to more accurately differentiate between temporary and chronic depressive conditions. Your guidance is greatly beneficial for our ongoing research endeavors.

5. Context in Existing Literature:

Thank you for your comments; it appears that you haven't raised any issues in this section.

6. Behavioral Factors and Health-Seeking Assumptions:

Thank you for your insightful comments regarding the assumptions made in our study regarding behavioral factors and health-seeking behaviors. We acknowledge that our current analysis does not directly support the behavioral assumptions due to the lack of relevant data in the NHANES database. We appreciate your guidance on this matter.

In response to your concern, we agree that future studies should delve deeper into this area. We plan to include data collection on participants' health-seeking behaviors, mental health management, and oral hygiene practices to better substantiate our claims. This will allow us to provide a more comprehensive understanding of the relationship between mental health and oral health.

Thank you again for your valuable feedback, which has illuminated a clear direction for our future research endeavors.

7. Unexplored Biological Pathways:

Thank you for your insightful comments. We appreciate the suggestion to explore the metabolic changes in obesity and their impact on inflammatory responses to depression. We will incorporate physiological markers like cortisol, cytokines, and adipokines in our future studies to better understand the interaction between depressive symptoms and periodontal health in the context of obesity.

**Reviewer #2:**

Introduction:

1. Thank you for your feedback. The sentence has been revised and split into two: “Obesity is defined as a condition in which the body mass index (BMI) exceeds 30 kg/m². Its prevalence has significantly increased worldwide. In 2008, 1.5 billion adults were classified as overweight, and by 2016, obesity rates had tripled.”
2. Thank you very much for your valuable suggestion! I have already stated the hypotheses (including the null and alternative hypotheses) in the last paragraph of the introduction, but the presentation may not have been sufficiently clear or explicit. I have now further emphasized and refined the statement of the hypotheses in the fourth paragraph of the introduction to ensure that readers can fully understand the research logic. Once again, I sincerely thank you for pointing this out and helping me improve the structure and clarity of the paper.

Methods:

1. Thank you for your careful observation! You are absolutely correct — it was a typo, and I did mean "attachment loss" instead of "alveoli." This error has been corrected in the “Outcome Variable” section of the Materials and Methods. I sincerely appreciate your attention to detail and your valuable feedback. Thank you!
2. Thank you very much for your valuable feedback! As you pointed out, the influencing factors of periodontitis are indeed complex and multifaceted. Due to the limitations of the NHANES database and practical constraints, it was not possible to include all potential confounding factors in this study. We have revised the discussion of the study's limitations accordingly in the seventh paragraph of the "Discussion" section. Thank you again for your insightful suggestions!

Discussion:

1. We sincerely appreciate your detailed feedback regarding the wording in our manuscript. We have thoroughly reviewed the Discussion section and revised the expression of "protective factor" to eliminate any potential misinterpretation. Instead, we now describe the relationship as a "negatively correlated with" in discussion sections. We believe this adjustment more accurately reflects our findings and avoids any misunderstanding. Thank you again for your rigorous review and professional advice, which have been invaluable in helping us improve the manuscript.
2. Based on your suggestions, we have made revisions and additions to the Introduction and Discussion sections of the manuscript. In these revisions, we more thoroughly analyzed the complex pathogenesis of periodontitis and provided a detailed discussion of the potential mechanisms underlying the negative correlation between periodontitis and depression in obese patients. Additionally, we emphasized the retrospective nature of the data and the limitations associated with questionnaire-based research in the Introduction, clearly stating their possible impact on the study results to ensure transparency.

We hope these revisions meet your expectations and further improve the quality of the manuscript. Thank you again for your thoughtful and constructive comments.
